# Finite-key security analyses on passive decoy-state QKD protocols with different unstable sources

**DOI:** 10.1038/srep15276

**Published:** 2015-10-16

**Authors:** Ting-Ting Song, Su-Juan Qin, Qiao-Yan Wen, Yu-Kun Wang, Heng-Yue Jia

**Affiliations:** 1 Department of Computer Science, College of Information Science and Technology, Jinan University, Guangzhou, 510632, China; 2 State Key Laboratory of Networking and Switching Technology, Beijing University of Posts and Telecommunications, Beijing, 100876, China; 3 School of Information, Central University of Finance and Economics, Beijing, 100081, China

## Abstract

In quantum communication, passive decoy-state QKD protocols can eliminate many side channels, but the protocols without any finite-key analyses are not suitable for in practice. The finite-key securities of passive decoy-state (PDS) QKD protocols with two different unstable sources, type-II parametric down-convention (PDC) and phase randomized weak coherent pulses (WCPs), are analyzed in our paper. According to the PDS QKD protocols, we establish an optimizing programming respectively and obtain the lower bounds of finite-key rates. Under some reasonable values of quantum setup parameters, the lower bounds of finite-key rates are simulated. The simulation results show that at different transmission distances, the affections of different fluctuations on key rates are different. Moreover, the PDS QKD protocol with an unstable PDC source can resist more intensity fluctuations and more statistical fluctuation.

Since the rapid development of quantum information, based on quantum mechanics and classical communication to achieve unconditional security, quantum cryptography has become the most important field of quantum information. The first proposed quantum key distribution (i.e., QKD) protocol, BB84 protocol^1^, was proved to be unconditionally secure under the perfect conditions^2^, which include perfect single-photon source, perfect measurement devices.

Also, the unconditional security of BB84 protocol is with the assumption “Alice and Bob exchange infinite pulses”. Actually, this assumption is inconsistent with the practical situation. The length of exchanged pulses is limited by the link duration constraints and memory resources on one side and efficiency on the other. In the use with satellites, the communication between the orbiting terminal and the ground station is restricted to a few minutes in the case of low-earth-orbit satellite^3^,^4^ or to about one hour for the medium-earth-orbit ones^5^. Furthermore, many researches on the finite-key BB84 protocols with decoy states^6^,^7^,^8^ showed that the finite pulses make an ideal remote QKD protocol into a short-distance but practical QKD protocol. Hence, for the practical application of QKD protocols, it is of crucial importance to analyze the security in the finite-key scenario, which should consider the affection of finite number of pulses.

Following the statistical fluctuation theory, the security of decoy-state QKD protocol is reflected by the lower bound of the key rate for the finite-key model. Suppose the eavesdropper operates the collective attack, the general formula for the lower bound of the final key rate with statistical fluctuations is


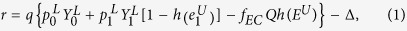


where *q* is the number of raw key per pulse received by Bob, 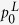 and 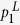 are the lower bounds of probability of the vacuum state and that of single-photon state sent from the signal source, 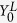 and 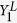 are the lower bounds of clicking rate for vacuum state and single-photon state, 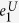 is the upper bound of error rate for single-photon state, *f*_*EC*_ reflects the deviation of practical error-correction codes from the Shannon limit, *Q* and *E*^*U*^ are the gain and the upper bound of the error rate for signal pulses generated to the final key, *h*(·) is the Shannon entropy, 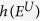 shows part of the information leaked to Eve during the error correction step, and the other part is included in Δ. Δ is comprised by the fluctuations of practical key rate induced by the “smoothing” level of min- and max-entropy 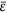
6,7,9, the failure probability of privacy amplification *ε*_*PA*_ and the failure probability of error correction *ε*_*EC*_. As refs 6,7,10 pointed out, Δ can be quantified by


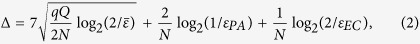


where *N* is the number of signal pulses sent from the source. The finite-key QKD protocol is called *ε*-secure, i.e., 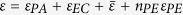, where *n*_*PE*_ is the number of parameters that must be estimated, and *ε*_*PE*_ is the failure probability of parameter estimation.

For the practical finite QKD protocols, there also exist two side channels. One follows the intensity modulator. In decoy-state QKD protocol, Alice modulates actively the weak laser into two weak coherent sources with different intensities. This is an elegant solution to implement the BB84 protocol, but some loopholes emerge in the practical implementations with Plug & Play systems^11^. So the finite passive-decoy-state (PDS) QKD protocols are thus desirable. Two, all the sources mentioned above are stable, i.e., the intensities are fixed. Actually, there exists the intensity fluctuations^12^ in practical sources. This is induced by the practical unstable sources^13^,^14^,^15^,^16^. No source can be perfectly stable. So does the sources in the finite PDS QKD protocols. That the sources are unstable means, at each time *i*, the intensity of source prepared by sender is 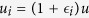 where *u* is the expected intensity of the source, and 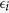 reflects the intensity fluctuation varying with the time *i*. The imperfection leaves a backdoor to eavesdropper, so the finite security of PDS QKD protocols with intensity fluctuations must be analyzed. In order to make sure the practical security of QKD protocols, this paper analyzes the finite securities of PDS QKD protocols with two unstable sources, type-II parametric down-convention (PDC) source^17^,^18^,^19^ and weak coherent pulses (WCPs)^20^,^21^.

## Results

This paper concerns on two kinds of fluctuations, intensity fluctuation and photon-number distribution fluctuation. Imprecise intensity control generates sources with intensity fluctuations. Because of the finite number of pulses in practical experiment, some parameters are to be with photon-number distribution fluctuations. We propose two finite PDS QKD protocols under different unstable sources, PDC source and WCPs. The process of two protocols and corresponding security analyses are as follows.

### Passive decoy-state QKD protocol with an unstable PDC source

Type-II PDC source could send out two pulses with the same number of photons at one time, but the polarizations of photons in the two pulses are different. Since the errors of PDC source^12^, especially the intensity fluctuations, the initial states sent from the source are different from the assumed states that Alice wants. Here, we only consider the affection of intensity fluctuations on the security of the QKD protocol with finite keys.

PDC source would send a pair of pulses at each integer time. If *N* pairs of pulses are sent, time *i* belongs to the integer set [1, *N*]. At time *i*, the intensity of PDC source is 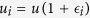 where *u* is the expected intensity and 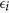 reflects the intensity fluctuations and its value belongs to the set 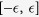 except with a small probability, so the initial state sent from the unstable PDC source at time *i* is 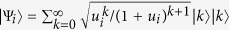 where the *k*-photon pulse is sent with the probability 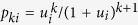. The two pulses go to different paths. The first pulse is sent to the polarization-independent detector located in Alice’s lab. The polarization of the second pulse is modulated according to Alice’s secret. Then the modulated pulse is sent to Bob through a high loss quantum channel. Bob receives the pulse, does the measurement with the basis randomly chosen by him, and records the result.

The transmitted part of quantum-state is finished, and the following part is the classical information post-process. After comparing the basis published by Bob, Alice tells Bob whether the bit is kept. If the bases used by Alice and Bob are same, the bit is kept, otherwise it is dropped. Then Alice and Bob repeat the previous steps many times until they get a string of bits. All the kept bits form the sifted bit sequence. According to the results of Alice’s detections, the sifted key bits are divided into two classes, the sifted bits with triggered and the sifted bits without triggered, as “trigger” and “non-trigger”^17^,^18^,^19^, respectively. Half of the sifted trigger pulses are the raw keys generated to the final keys, and the other half is used to estimate the bit error rate. The sifted non-trigger pulses are used to estimate the phase error rate.

The security analysis of the protocol is as follows. The trigger pulses and the non-trigger pulses are classified passively according to the results of corresponding pulses at Alice’s detector. Before the corresponding pulses arrived at her detector, Alice has no information about the classification of the pulses, so the eavesdropper has no information on the pulses before they arrive at Alice’s detectors, either. Thus the classifying method is passive and secure to any powerful eavesdropper. The security of the following steps in PDS QKD protocol with an unstable PDC source is equivalent to that of standard BB84 protocol with decoy method. The protocol introduced above is secure.

Now we show that how to get the lower bound of the finite-key rate with the affections of intensity fluctuations and photon-number distribution fluctuations. In the PDS QKD protocol with an unstable PDC source, whatever the intensity fluctuation is at each time, Alice knows the maximum and the minimum of the expected intensity, and the two boundary values should be estimated with statistical fluctuation. The failure probability of parameter estimation is denoted as *ε*_*PE*_. According to the statistical fluctuation of photon-number distribution, we can get the lower bound of the key rate for the PDS QKD protocol. In the following, we give the particular analysis on that how to get the lower bound of the finite-key rate with the affections of intensity fluctuations and statistical fluctuations.

In our analysis, we assumed the worst case that eavesdropper knows exactly the intensity fluctuation of each pulse, as Refs 13, 14, 15, 16, so the clicking rate of *k*-photon pulses at Bob’s detector and the error rate of *k*-photon pulses are both changed with time *i*, which is different from earlier work^6^,^7^,^8^. Thus the number of sifted trigger pulses received by Bob is 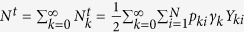, and the number of sifted non-triggered pulses received by Bob is 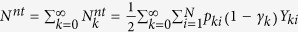, where *N* is the total number of pulses sent from the source, *p*_*ki*_ is the probability that Alice sends a pair of *k*-photon pulse to Bob at time *i*, *γ*_*k*_ is the clicking rate of Alice’s detector for *k*-photon pulse, and *Y*_*ki*_ is the clicking rate of Bob’s detectors at time *i* when Alice sends Bob a *k*-photon pulse. Note that here we suppose that the clicking rate *γ*_*k*_ doesn’t vary with time. Denote the number of bit errors and that of phase errors checked by Alice and Bob as *n*^*t*^ and *n*^*nt*^ respectively. There have 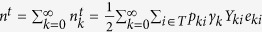 and 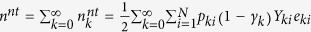, where set *T* has *N/*2 elements randomly chosen from the index set [1, *N*], and *e*_*ki*_ is the error rate of Bob’s sifted results when Alice sends Bob a *k*-photon pulse at time *i*.

Generally speaking, the probability of *k*-photon trigger pulses is different from that of *k*-photon non-trigger pulses, thus the sifted trigger pulses and the sifted non-trigger pulses could give the lower bound of the key rate by the economic estimated method^15^. But here has another problem: any expect value as 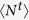, 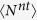, 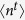, 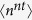, and their bound values with intensity fluctuation 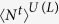, 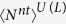, 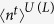, 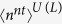 do not be applied in practice directly. Practical implementation needs the practical values of these parameters, which should consider the influence of finite-number of pulses. We give the lower bounds and the upper bounds of these parameters by statistical fluctuation theory^8^, as follows,


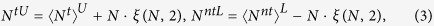



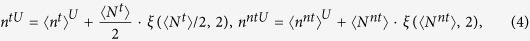


where 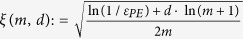. Following Eq. (1), the lower bound of key rate for this PDS QKD protocol will be


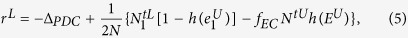


where 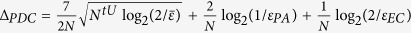, 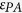 and 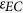 are the failure probabilities of privacy amplification and the error correction respectively, 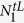 is the lower bound of 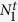, 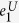 is the upper bound of *e*_1*i*_, and 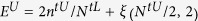 is the upper bound of phase error rate of raw key. The rigorous proof of Eq. (5) is in Methods. Note that the lower (upper) bound of the expected value 〈*〉^*L*(*U*)^ mentioned here represents the lower (upper) bound of parameter *with intensity fluctuation only, and the lower (upper) bound of the observed value *^L(*U*)^ represents the lower (upper) bound of parameter *with both intensity fluctuation and statistical fluctuation.

### Passive decoy-state QKD protocol with unstable WCPs

**Figure 1:**
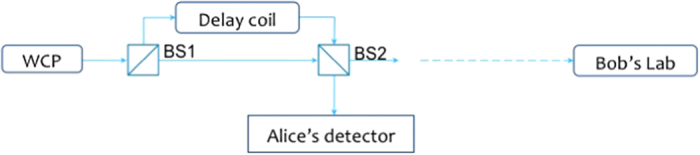
The apparatuses in PDS QKD protocol with an unstable WCP source.

The requirement of PDS method is to have correlations between the photon number statistics of two signals. Curty *et al.*^22^ pointed out that the photon numbers of outgoing pulses are classically relevant when two phased randomized WCPs interfere at a beam splitter. Based on this, we propose a PDS QKD protocol with unstable phased randomized WCPs, which could be realized easily by linear optical components. The setups are shown in Fig. 1.

The protocol is described below. Alice prepare one phase randomized weak coherent father source whose expected intensity is *u*. Due to intensity fluctuations, the output state from the father source at time *i* is 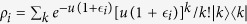, where 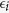 gives the fluctuation of intensity, and its value belongs to 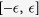 except with a small probability *ε*_*PE*_. Through the beam splitter BS1, the pulses are separated into two parts, one to the upper path, and the other to the lower path. The upper part would interfere with the lower part on another beam splitter BS2 after the upper part transmitted through a delay coil. And there is a polarization independent detector at one of the outputs of BS2 which is located in Alice’s lab. The pulses coming out from the other output of BS2 are sent to the receiver Bob. After receiving the pulses, Bob does the measurement with randomly chosen bases. The quantum distribution is finished, and the following process is classical process, including sifting data, error corrections and privacy amplification. Comparing the bases for each pulse, Alice and Bob keep the bits with same bases, and the bit sequence becomes the sifted key. To be concerned, the pulses sent to Bob are also passively classified into two kinds, “trigger” and “non-trigger”, according to the results of the detector in Alice’s lab. During the error correction process, all the sifted non-trigger pulses estimate the phase error rate, and half of the sifted trigger pulses estimates the bit error rate. According to the privacy amplification, the other half of the sifted trigger pulses is generated to the final key.

Note that there should be an intensity modulation (IM) before Bob’s Lab, and its operation is correlated with the length of delay coil. Denote the state of the upper path and that of the lower path after BS1 at time *i* as *ρ*_1*i*_ and *ρ*_2*i*_. We can adjust the length of the delay coil on the upper path to make the pulse *ρ*_1,*i*−1_ sent at time *i* − 1 and the pulse *ρ*_2*i*_ sent at time *i* interfere at BS2. In order to eliminate all the possible correlations between the results hold by Bob, IM should block the pulses arrived at even time or at odd time. Now the signal states that go to Bob are guaranteed to be tensor products of mixtures of Fock states.

The protocol would resist against the side channels on modulators and detectors. Suppose BS1 is with transitivity rate *t*_1_, the state of the upper path and that of the lower path at time *i* are as follows:


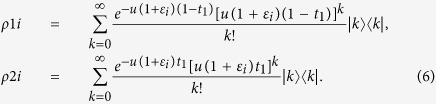


After through BS2 with transitivity rate *t*_2_, the joint probability that *k*-photon pulse is sent to Bob and the *m*-photon pulse is sent to Alice’s detector at time *i* is denoted as 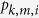, so the probability that *k*-photon pulse is sent to Bob at time *i* is denoted as 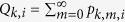. If Alice’s detector at the end of one output of BS2 responses with the probability *γ*_*m*_ for *m*-photon pulse, at time *i* the probability of non-triggered *k*-photon pulse sent to Bob is 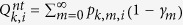, and the probability of triggered *k*-photon pulse sent to Bob is 
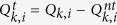
. At time *i*, the distributions 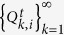 and 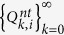 are different but classical correlated. The pulses sent to Bob are passively classified into two classes, “trigger” and “non-trigger”. During the pulses transmitted through the quantum channels, eavesdropper would get no information about the classification, no matter which kind of attack operated by her. Thus the PDS QKD protocol is secure without any information leakages.

Furthermore, same as the PDS QKD protocol with an unstable PDC source, whatever intensity fluctuates is at each time in the PDS QKD protocol with unstable WCPs, Alice only know the maximum and the minimum of the expected intensity. The failure probability of intensity-fluctuation estimation is also defined as 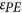, then according to Eq. (1) we would get the lower bound of the key rate.

Suppose that the number of the pulses sent from the source is *N*, so time *i* is the integer in the set 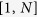, and IM blocks all the pulses sent at odd time. Same as the process of the PDS QKD protocol with an unstable PDC source, in this with unstable WCPs, both the clicking rate of *k*-photon pulses at Bob’s side *Y*_*ki*_ and the error rate of *k*-photon pulses *e*_*ki*_ are correlated with time *i*, so the number of sifted trigger pulses and that of sifted non-trigger pulses received by Bob, respectively, are


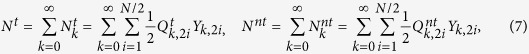


where half of the sifted trigger pulses estimates the bit error rate, all the odd pulses are blocked by IM. The number of bit errors and that of phase errors are


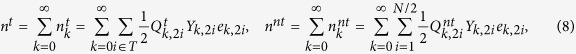


where the set *T* has *N*/4 elements chosen randomly from the index set [1, *N*/2]. The expected values of above parameters can be calculated without considering the statistical fluctuations, and with statistical fluctuations we can get the practical values of these parameters. The relationship between the expected value and the practical values are same as Eq. (3). Based on the bounds of practical values 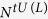, 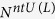, 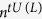, 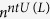, the lower bound of key rate is received


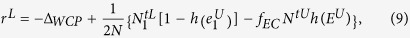


where 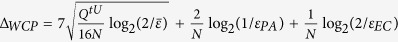, 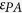 and 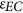 are the failure probabilities of privacy amplification and the error correction respectively, 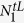 is the lower bound of 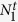, 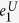 is the upper bound of 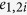, and 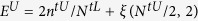 is the upper bound of phase error rate of raw key. As described earlier, 〈*〉^*L*(*U*)^ denotes the lower (upper) bound of the expected value, and *^L(*U*)^ represents the lower (upper) bound of the observed value. Note that the formula of failure probability applied in this work is actually for a stable source while here we are studying the performance of QKD with an unstable. This issue should be further studied in the future. The rigorous process to get the parameters in Eq. (9) is in Methods.

## Discussion

In this section, we will discuss the asymptotic finite-key rate with the unstable PDC source and that with the unstable WCPs. For this purpose, we firstly establish the models for quantum setups. In both PDS QKD protocols, the model for Alice’s detector and that for quantum channel including Bob’s detectors are same. Suppose Alice’s detector is with efficiency *η*_*A*_ and dark count rate *d*_*A*_. The detector is triggered by the *k*-photon pulse with the rate 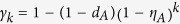, so the non-triggered rate is 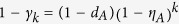. Furthermore, we establish the model for the expected value 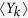, and this simplification wouldn’t affect the accuracy of the following discussion because the finite-key rates are related with the boundary values. The *k*-photon pulse that Alice sends to Bob would click on Bob’s detectors with the expected probability 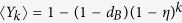, where *d*_*B*_ is the dark count rate of Bob’s detectors, 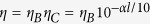 is the total transmission rate, *η*_*B*_ is the efficiency of Bob’s detector, *η*_*C*_ is the transmittance of quantum channels, *α* (dB/km) is the lossy rate of quantum channels, and *l* (km) is the transmission distance. Then the different models and simulation results for two PDS QKD protocols are as follows.

### Model for an unstable PDC source

Based on the model of detectors described before, we would get the gains and error rates for the PDS QKD protocol with an unstable PDC source


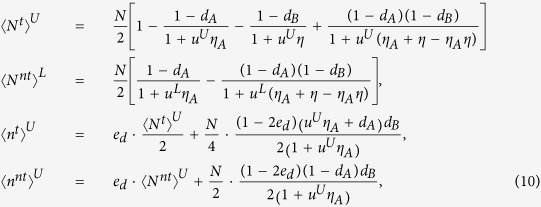


where *e*_*d*_ is the error rate of Bob’s detectors. The upper bounds are all obtained when the intensity of the source reaches the maximum value *u*^*U*^ with a large probability. Correspondingly, the lower bound is received with minimum value of the intensity *u*^*L*^.

### Model for an unstable WCP source

Following the models of quantum setups and the protocol in Results, we obtain the probability that Alice’s detectors at time *i* are non-triggered, i.e., 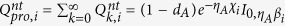, where 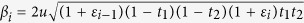, 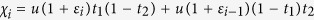, 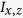 represents the modified Bessel function of first kind and is defined as 
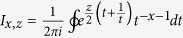
. Supplementary Material shows the calculating process that how to get 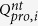 and the upper bounds of some expect values except the followings,


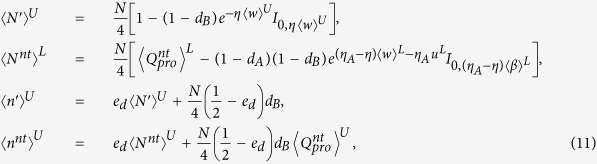


where 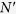 is the number of pulses received by Bob, 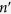 is the number of error bits in the pulses received by Bob, and


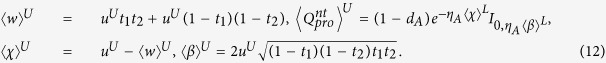


### Simulation Results

We simulate the finite-key rates for both of PDS QKD protocols based on the models. The values of some experimental parameters in Eqs (10,11) are supposed to be as follows. Assume Alice’s detector is a typical silicon avalanche photodiode with *d*_*A*_ = 3.2 * 10^−7^ and 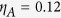. Other experimental parameters are chosen as Refs 10,23, 24, 25, 26, 27, 28, 29, 30: the detection efficiency of Bob’s detectors 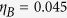, the dark count rate of Bob’s detectors *d*_*B*_ = 1.7 * 10^−6^, the loss coefficient of the quantum channel 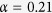, the error rate of the optical system 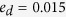, and the efficiency of error correction code *f* = 1.22. The sum of all failure probabilities is 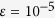, and the failure probability of parameter estimation is 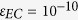. Followed the equations about the lower bounds of finite-key rates, the other parameters are optimized to maximize the finite-key rates, including the expected intensity *u*, the failure probabilities 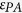, 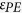, the transmission rates of beam splitters *t*_1_, *t*_2_.

**Figure 2:**
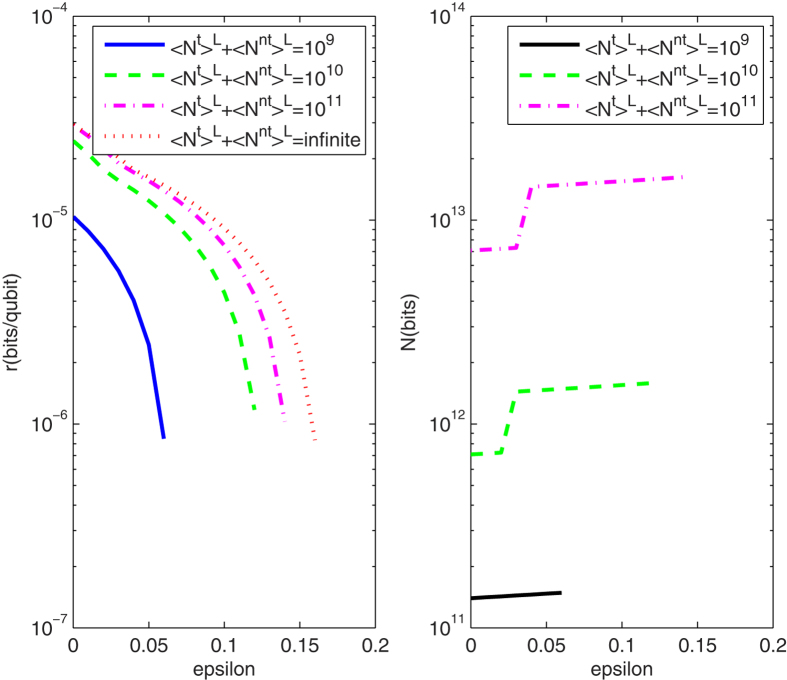
(Left) The lower bounds of key rates for PDS QKD protocols with an unstable PDC source under different numbers of pulses that Bob receives, when the transmission distance is fixed as 50 km; (Right) The corresponding optimal number of pulses sent from the PDC source with the transmission distances 50 km, when the numbers of pulses received by Bob is 10^9^, 10^10^, and 10^11^.

**Figure 3:**
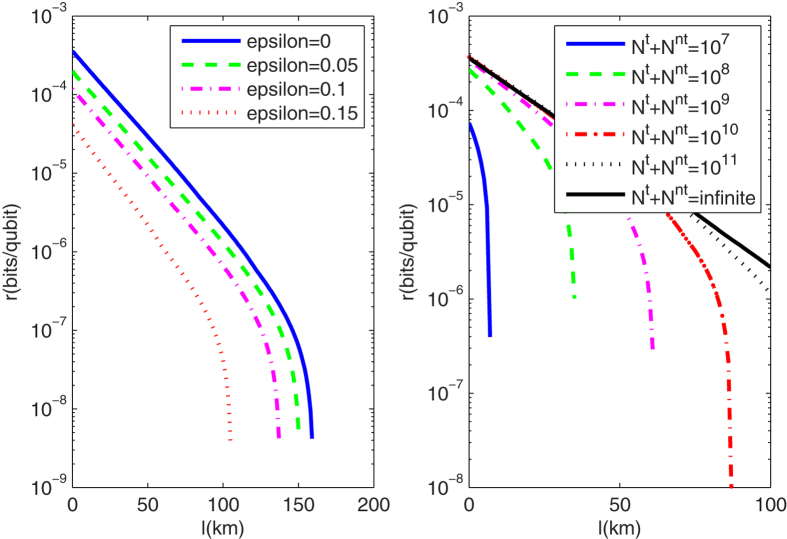
(Left) The lower bounds of key rates for PDS QKD protocols when the intensity fluctuations are 0, 0.05, 0.1, 0.15 and the number of pulses are infinite; (Right) The lower bounds of key rates for PDS QKD protocols with a stable PDC source under different numbers of pulses that Bob receives.

Figure 2 (Left) shows the lower bounds of key rates for PDS QKD protocols with a PDC source under the conditions that Bob receives 10^9^, 10^10^, 10^11^ and infinite pulses, when the transmission distance is fixed as 50 km. The corresponding optimal number of pulses sent from the PDC source is shown in Fig. 2(Right). From the curves in Fig. 2(Left), we know that the lower bounds of key rates without intensity fluctuations are all with the order of 10^−5^. With the intensity fluctuations increasing, the key rates decrease, and the finite-key rates approach to the infinite-key rates with the number of pulses increasing too much. Furthermore, in order to find the main affection on key rates^15^, we give the lower bounds of key rates without intensity fluctuations or without photon-number-distribution fluctuation in Fig. 3. When there is no photon-number-distribution fluctuation, the protocol has positive key rates with intensity fluctuations *ε* < 0.2, and the key rates without intensity fluctuations are about 10 times than the key rates with *ε* = 0.15 if the transmittance distance is shorter than 100 km. Moreover, if the PDC source is stable, the lower bound of finite-key rates in the PDS QKD protocol are positive with the number of pulses more than 10^7^ in Fig. 3(Right). Compared two figures, if the transmittance distance is shorter than 10 km, as the number of pulses increasing from 10^7^ to its 9 times, the key rates increase less than that as the intensity fluctuation decreasing from *ε* = 0.15 to its 9 times, but that is to be contrary if the transmittance distance is farther than 10 km. So, the photon-number-distribution fluctuations introduced by finite-number of pulses are the main cause, if the distance between communication parties is farther than 10 km. Within 10 km, the intensity fluctuation is the main cause. Note that this is deduced within the scope of the resistible intensity fluctuations and the scope of the number of pulses that can yield secure keys in the protocol.

**Figure 4:**
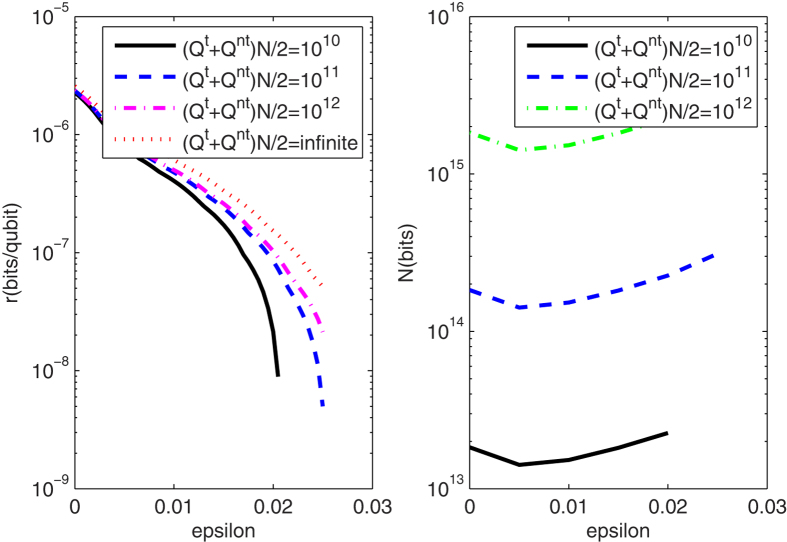
(Left) The lower bounds of key rates for PDS QKD protocols with unstable WCPs under different numbers of pulses that Bob receives, when the transmission distance is fixed as 50 km; (Right) The corresponding optimal number of weak coherent pulses sent from the source with the transmission distances 50 km.

**Figure 5:**
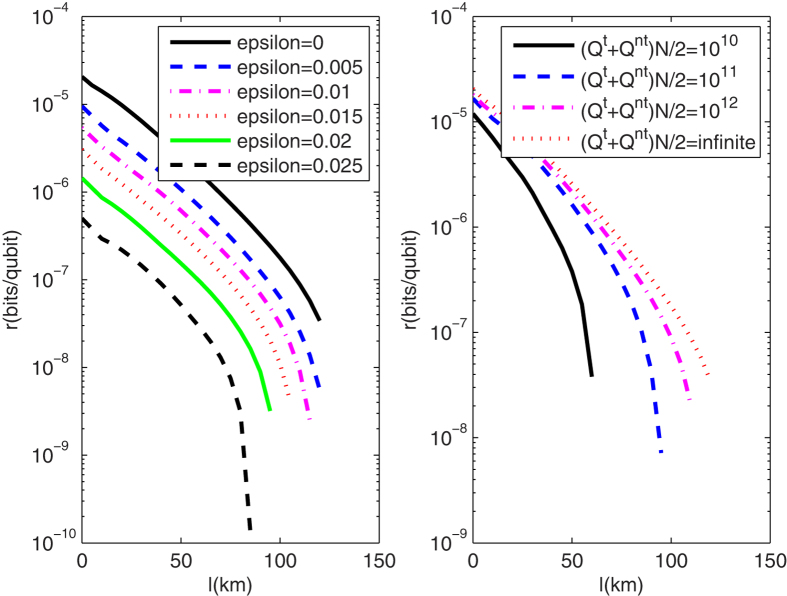
(Left) The lower bounds of key rates for PDS QKD protocols when the intensity fluctuations are 0, 0.005, 0.01, 0.015, 0.02, 0.025 and the number of pulses are infinite; (Right) The lower bounds of key rates for PDS QKD protocols with stable WCPs under different numbers of pulses that Bob receives.

For the PDS QKD protocol with unstable WCPs, we simulate the key rates when the numbers of pulses received by Bob are 10^10^, 10^11^, 10^12^, infinite, and the transmission distance is fixed as 50 km in Fig. 4(Left). The finite-key rates are arbitrarily close to the key rates with infinite number of pulses as the number of pulses sufficiently close to infinite. If Bob receives less than 10^10^ pulses, secure keys can not be obtained. Figure 4(Right) shows the corresponding optimal number of pulses sent from Alice when the transmission distance is 50 km and Bob receives 10^10^, 10^11^, 10^12^ pulses. For the curves in Fig. 4(Right), the least number of pulses sent from Alice is 10^13^, which needs that 10G High-speed QKD system runs more than 1000 seconds without stop. We also describe the affections of intensity fluctuations on key rates and that of the photon-number-distribution fluctuations on key rates in Fig. 5(Left) and Fig. 5(Right) respectively. Within the resistible intensity fluctuations, Fig. 5(Left) shows that for different intensity fluctuations the decreasing velocities of the key rates at same transmission distance is much same, but the curves in Fig. 5(Right) do not have the same characters. Comparing the two figures in Fig. 5, we know that if the transmittance distance is shorter than 50 km, as the number of pulses increasing from 10^10^ to its 9 times, the key rates are changed less than that with the intensity fluctuation decreasing from *ε* = 0.15 to its 9 times. Therefore, if the distance between communication parties is farther than 50 km, the photon-number-distribution fluctuations are the main affections on the key rates. Within 50 km, the intensity fluctuation is changed into the main cause. This is deduced within the scope of the resistible intensity fluctuations and the number of pulses that can yield positive key-rates in the protocol.

### Comparison

We study the applications of two different sources in PDS QKD protocols. In both of protocols, the finite-key rates are all affected by intensity fluctuations and the photon-number-distribution fluctuations. Comparing Fig. 2(Left) and Fig. 4(Left), we know that while the protocol with an unstable PDC source even can resist intensity fluctuations with *ε* < 0.15, the PDS QKD protocol with unstable WCPs resist much less intensity fluctuations, and it cannot generate secure key with the number of pulses less than 10^10^. With the same intensity fluctuations and the same transmission distances, the finite-key rates of PDS QKD protocol with PDC source are much higher than that with WCPs. Moreover, for large scale of transmission distances, the PDS QKD protocol with unstable PDC source is mainly affected by the photon-number-distribution fluctuations, which are in the control of communicating parties.

## Methods

It is difficult to obtain the values of 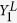 and 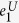 in the key rates of Eq. (1), but the authors in Refs 14, 15, 16 have introduced many methods to obtain the two values. The methods are general and can be applied to any fluctuation of parameters in the source state. Following the models established in Discussion Section, we will show how to get each parameter in the formula of final finite-key rate. Both the methods are inspired by the idea in Refs 14, 15, 16.

### Finite-key rates for an unstable PDC source

In order to get the lower bound of the sifted trigger number of the single-photon pulses 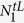 and the upper bound of error rate of the single-photon pulses 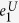, we introduce the influences of statistical fluctuations and intensity fluctuations on some expected parameters.

The lower bound and the upper bound of the source intensity *u*_*i*_ are 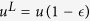 and 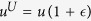, respectively. In the following, 〈*〉^*L*(*U*)^ is the lower bound (the upper bound) of the expected value of parameter *, and *^L(*U*)^ is the lower bound (the upper bound) of the observed value of parameter *.

According to ref. 15, the lower bound of 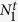 is


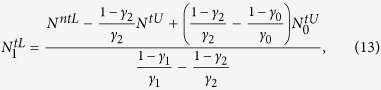


if there exists 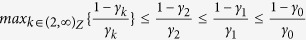, where


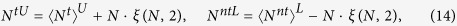



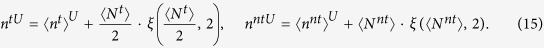


And also we can get the upper bound of the clicking number of sifted trigger vacuum pulses 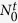 and the error rate of single-photon pulse *e*_1*i*_


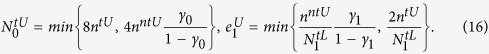


Followed the upper bound of error rate 
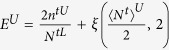
, the lower bound of finite-key rate is obtained


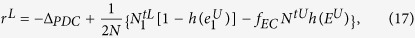


where 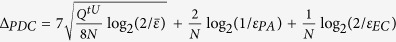. Note that the key rate without photon-number-distribution fluctuations in Fig. 2(Left) is easily obtained from above discussion, i.e.,


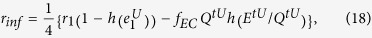


where


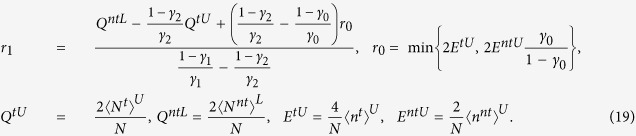



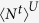
, 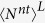, 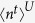 and 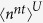 are shown in Eq. (10).

### Finite-key rates for an unstable WCP source

In order to make our paper complete, we apply the results in ref. 13 to obtain the values of 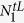 and 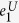 here, through replacing the observed values by the expected values of each quantities there^13^.

The lower bound of 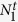 is


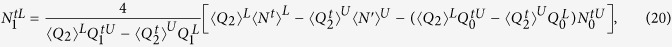


under the conditions 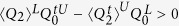 and 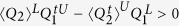, where


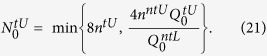


Similarly, we can obtain the upper bound of error rate of single-photon pulses, i.e.,


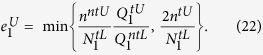


Note that in the right hand side of Eq. (20), all quantities are expected values, while in the practical implementation, they are actually observed values.

Now the final finite-key rate is received as follows:


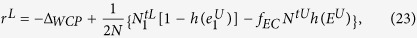


where 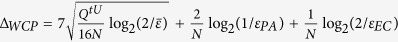. According to the discussion, we also can obtain the key rate without photon-number-distribution fluctuations, i.e.,


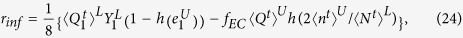


where 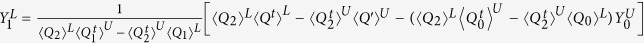, 
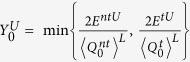
, 
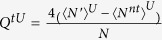
, 
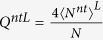
, 
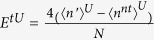
, 
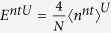
. 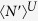, 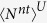, 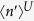 and 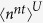 are from in Eq. (11). The simulation results of infinite-key rates are shown in Fig. 4 (Left).

## Additional Information

**How to cite this article**: Song, T.-T. *et al.* Finite-key security analyses on passive decoy-state QKD protocols with different unstable sources. *Sci. Rep.* **5**, 15276; doi: 10.1038/srep15276 (2015).

## Supplementary Material

Supplementary Information
