# Supplementary material for: Finite-key security analyses on passive decoy-state QKD protocols with different unstable sources
Source: Sci Rep. 2015 Oct 16;5:15276. doi: 10.1038/srep15276 (PMC4607920; doi:10.1038/srep15276)
Supplement: Supplementary Information [file srep15276-s1.pdf]

Finite-key security analyses on passive decoy-state QKD protocols with different unstable sources

Ting-Ting Song, Su-Juan Qin, Qiao-Yan Wen, Yu-Kun Wang, and Heng-Yue Jia

July 10, 2015

Supplementary Material

After through BS2 with transitivity rate t_2, the joint probability at time i that k-photon pulse is sent to Bob and m-photon pulse is sent to Alice's detector is

$$p_{k,m,i} = \frac{v_i^{k+m} e^{-v_i}}{k!m!} \frac{1}{2\pi} \int_0^{2\pi} r_i^k (1-r_i)^m d\theta, \tag{1}$$

where θ is the phase difference between the two pulses inputting BS2 at the same time, and

$$\begin{aligned} v_i &= u(1+\epsilon_{i-1})(1-t_1) + u(1+\epsilon_i)t_1, \\ r_i &= \frac{1}{v_i}\left[u(1+\epsilon_{i-1})(1-t_1)(1-t_2) + u(1+\epsilon_i)t_1t_2 + \beta_i \cos\theta\right]. \end{aligned} \tag{2}$$

According to the models of quantum setups described in Discussion Section, if the m-photon pulse will click at Alice's detector with the probability γ_m, Bob's detectors are non-triggered by k-photon with the probability

$$Q_{k,i}^{nt} = \sum_{m=0}^{\infty} p_{k,m,i}(1-\gamma_m) = (1-d_A)\frac{v_i^k e^{-\eta_A v_i}}{k!} \frac{1}{2\pi} \int_0^{2\pi} r_i^k e^{-(1-\eta_A)v_i r_i} d\theta. \tag{3}$$

Introducing the modified Bessel function of first kind $I_{x,z} = \frac{1}{2\pi i}\oint e^{\frac{z}{2}(t+\frac{1}{t})} t^{-x-1} dt$, we can get the following values Q_k^{nt},

$$\begin{aligned} Q_{0,i}^{nt} &= \tau_i I_{0,(1-\eta_A)\beta_i}, \\ Q_{1,i}^{nt} &= \tau_i (w_i I_{0,(1-\eta_A)\beta_i} - \beta_i I_{1,(1-\eta_A)\beta_i}), \\ Q_{2,i}^{nt} &= \frac{\tau_i}{4}[(2w_i^2 + \beta_i^2) I_{0,(1-\eta_A)\beta_i} - 4 w_i \beta_i I_{1,(1-\eta_A)\beta_i} + \beta_i^2 I_{2,(1-\eta_A)\beta_i}, \end{aligned} \tag{4}$$

where $\tau_i = (1-d_A)e^{-\eta_A u_i - (1-\eta_A)w_i}$, $w_i = u_i[t_1t_2 + (1-t_1)(1-t_2)]$, and $\beta_i = 2u_i\sqrt{(1-t_1)(1-t_2)t_1t_2}$.

The probability that the pulse sent to Bob has k photons is

$$Q_{k,i} = \sum_{m=0}^{\infty} p_{k,m,i} = \frac{v_i^k}{k!}\frac{1}{2\pi}\int_0^{2\pi} r_i^k e^{-v_i r_i} d\theta. \tag{5}$$

Some special values can be calculated with the modified Bessel function of first kind $I_{x,z}$

$$\begin{aligned} Q_{0,i} &= e^{-w_i} I_{0,\beta_i}, \\ Q_{1,i} &= e^{-w_i}(w_i I_{0,\beta_i} - \beta_i I_{1,\beta_i}), \\ Q_{2,i} &= e^{-w_i}\left[(\frac{w_i^2}{2} + \beta_i^2) I_{0,\beta_i} - w_i \beta_i I_{1,\beta_i} + \beta_i^2 I_{2,\beta_i}\right]. \end{aligned} \tag{6}$$

Furthermore, the probabilities $Q_{ki}^{t} = Q_{ki} - Q_{ki}^{nt}$ can be easily obtained.
